# Variability in antivenom neutralization of Mexican viperid snake venoms

**DOI:** 10.1371/journal.pntd.0012152

**Published:** 2024-05-08

**Authors:** Alid Guadarrama-Martínez, Edgar Neri-Castro, Leslie Boyer, Alejandro Alagón

**Affiliations:** 1 Departamento de Medicina Molecular y Bioprocesos, Instituto de Biotecnología, Universidad Nacional Autónoma de México, Cuernavaca, Morelos, México; 2 Facultad de Ciencias Biológicas, Universidad Juárez del Estado de Durango, Gómez Palacio, Durango, México; 3 Department of Pathology, University of Arizona, Tucson, Arizona, United States of America; Federal University of Roraima, BRAZIL

## Abstract

**Background:**

Each year, 3,800 cases of snakebite envenomation are reported in Mexico, resulting in 35 fatalities. The only scientifically validated treatment for snakebites in Mexico is the use of antivenoms. Currently, two antivenoms are available in the market, with one in the developmental phase. These antivenoms, produced in horses, consist of F(ab’)_2_ fragments generated using venoms from various species as immunogens. While previous studies primarily focused on neutralizing the venom of the *Crotalus* species, our study aims to assess the neutralization capacity of different antivenom batches against pit vipers from various genera in Mexico.

**Methodology:**

We conducted various biological and biochemical tests to characterize the venoms. Additionally, we performed neutralization tests using all three antivenoms to evaluate their effectiveness against lethal activity and their ability to neutralize proteolytic and fibrinogenolytic activities.

**Results:**

Our results reveal significant differences in protein content and neutralizing capacity among different antivenoms and even between different batches of the same product. Notably, the venom of *Crotalus atrox* is poorly neutralized by all evaluated batches despite being the primary cause of envenomation in the country’s northern region. Furthermore, even at the highest tested concentrations, no antivenom could neutralize the lethality of *Metlapilcoatlus nummifer* and *Porthidium yucatanicum* venoms. These findings highlight crucial areas for improving existing antivenoms and developing new products.

**Conclusion:**

Our research reveals variations in protein content and neutralizing potency among antivenoms, emphasizing the need for consistency in venom characteristics as immunogens. While Birmex neutralizes more LD_50_ per vial, Antivipmyn excels in specific neutralization. The inability of antivenoms to neutralize certain venoms, especially *M*. *nummifer* and *P*. *yucatanicum*, highlights crucial improvement opportunities, given the medical significance of these species.

## Author summary

The annual incidence of snakebite in Mexico is about 3,800 cases, leading to 35 fatalities. Therefore, antivenoms, which are the primary treatment, are crucial. Two Mexican antivenoms are in therapeutic use, and a third is in development; all are made from horse-derived antibody fragments. Our study assesses antivenom’s effectiveness in neutralizing the venom of several vipers in Mexico. We tested different antivenom batches, finding differences in neutralization capacity. Surprisingly, the *Crotalus atrox* venom is not well neutralized, and all antivenoms struggle against those of *Metlapilcoatlus nummifer* and *Porthidium yucatanicum*. Altogether, our work reveals crucial areas for antivenom improvement, stressing the need for better antivenoms, mainly due to the medical relevance of these species.

## Introduction

Snakebite envenomation is a recognized global health issue classified as a Neglected Tropical Disease (NTD) by the World Health Organization [1]. Worldwide, the highest estimates indicate an annual occurrence of envenomations ranging from 1,841,000 to 2,682,500, resulting in 81,000 to 138,000 deaths and over 400,000 survivors with debilitating sequelae [2,3]. In Mexico, the available epidemiological information on snakebite accidents is inadequate. According to the National Institute of Statistics and Geography (INEGI) and the National System of Epidemiological Vigilance (SINAVE), an average of 3,893 bites and 35.5 deaths were reported annually from 2003 to 2019 [4]. However, these data sources underestimate the true epidemiological burden due to three main factors: 1) many affected individuals in communities seek traditional treatments from healers or shamans, which goes unaccounted for in official records; 2) there is a lack of information regarding survivors with long-term sequelae; and 3) numerous public health facilities do not report data to SINAVE [4].

Although specific information for Mexico is lacking, it is plausible that, similar to the rest of the Americas, over 90% of snakebite envenomations are caused by snakes of the family Viperidae (viperids) due to their prevalence [5–9].

In Mexico, there are ten genera of viperids (*Agkistrodon*, *Bothriechis*, *Bothrops*, *Cerrophidion*, *Crotalus*, *Metlapilcoatlus*, *Mixcoatlus*, *Ophryacus*, *Porthidium*, and *Sistrurus*), comprising a total of 74 species. The venom of vipers is composed of three dominant protein families: phospholipases A_2_ (PLA_2_s), snake venom metalloproteases (SVMPs), and snake venom serine proteases (SVSPs). Together, these families account for about 77% of the venom proteome, and the remaining 22% is made up of other protein families: L-amino acid oxidases (LAAO), cysteine-rich secretory proteins (CRISP), C-type lectins (CTL), disintegrins (DIS) and natriuretic peptides (NP) [10,11]. In addition to the inherent intergeneric and interspecific diversity in venom composition, reports have correlated variation in the venom of viperids with diet [12–15], age of the organism [15–20], and sex [21]. These factors multiply the number of venoms with different characteristics and make the challenge for public health response even more complex.

Two commercial antivenoms are currently available to treat viperid snakebite envenomation in Mexico (Antivipmyn and Faboterápico Polivalente Antiviperino, hereafter, Birmex), and a third antivenom is undergoing experimental development (developed by Inosan Biopharma). All three are derived from equine sources and utilize F(ab’)_2_ fragments as their active components, but they are based on different venom immunogens. One of the commercial antivenoms, Birmex, is produced by Laboratorios de Biológicos y Reactivos de México S.A. de C.V., a state-owned company with a history of approximately 90 years in antivenom production [22,23]. Birmex employs a mixture of *Crotalus basiliscus* and *Bothrops asper* venoms as immunogen to manufacture around 24,000 vials per year of their polyspecific antivenom. Another commercially available antivenom is Antivipmyn, produced by Laboratorios Silanes S.A. de C.V. This antivenom uses venom from *B*. *asper* and *C*. *simus* as immunogens. However, as Antivipmyn was registered before the update in the *C*. *simus* taxonomy status [24,25], the venoms of *C. mictlantecuhtli*, *C. tzabcan*, and *C. culminatus* are likely included in its immunization mix, too. Additionally, Inosan Biopharma is developing an experimental antivenom called Inoserp, using a mixture of venoms from *B*. *asper*, *C*. *basiliscus*, *C*. *mictlantecuhtli*, *C*. *culminatus*, *C*. *tzabcan*, *C*. *atrox*, *C*. *molossus*, *C*. *s*. *scutulatus*, *C*. *s*. *salvini*, *Agkistrodon bilineatus*, and *Sistrurus catenatus* as immunogens.

While the production of antivenoms in Mexico has a longstanding history and is regarded as among the world’s best, there is currently a lack of studies assessing their neutralizing capacity against viperid species outside of the *Crotalus* genus. We aim to analyze the recognition and neutralization capabilities of the two commercial antivenoms and the experimental antivenom against venom from various genera of Mexican pit vipers.

## Methodology

### Ethics statement

Mice employed in this study were obtained from the Instituto de Biotecnología—Universidad Nacional Autónoma de México bioterium. These animals were kept at 12:12 h in a light-dark cycle with water, and food *ad libitum*. All procedures with animals followed the protocols established by the Bioethics Committee of the Instituto de Biotecnología—Universidad Nacional Autónoma de México and were approved under project number 345.

### Samples of Venoms and Antivenoms

Venoms were obtained from the venom bank of Herpetario Cantil at the Instituto de Biotecnología—Universidad Nacional Autónoma de México. To ensure a diverse range of venoms, we analyzed samples from nine viperid species representing seven distinct genera: *Agkistrodon bilineatus*, *Bothrops asper*, *Cerrophidion tzotzilorum*, *Crotalus atrox*, *Crotalus basiliscus*, *Crotalus mictlantecuhtli*, *Metlapilcoatlus nummifer*, *Ophryacus sphenophrys*, and *Porthidium yucatanicum*. All these species are found in Mexico, with five being endemic. For the neutralization tests, we employed three different lots of Antivipmyn, three lots of Birmex, and two lots of Inoserp. All experiments were conducted within the expiration dates of the respective lots (Table 1).

**Table 1:** Lots of the antivenoms included in the present study.

Antivenom	Lots	Expiration date
Antivipmyn	B-6J-31	August-2020
	B-8K-31	September-2022
	B-8H-34	June-2022
Birmex	FV045A	February-2021
	FV043A	September-2020
	FV044A	October-2020
Inoserp	8805181002	October-2020
	8805181003	November-2020

### Determination of protein concentration

The protein concentration of antivenoms was determined spectrophotometrically, assuming that an A_280nm_ of 1.4 equaled 1 mg/mL [26]. Protein determination of the venom samples was performed using the Pierce BCA Protein Assay kit (Thermo Scientific, Rockford, IL, US) following the manufacturer’s instructions and using bovine serum albumin (BSA) standards.

### Electrophoretic profiles

Electrophoresis was carried out on 12.5% polyacrylamide gels in the presence of sodium dodecyl sulfate (SDS-PAGE) [27] using a Hoefer Tall Mighty Small SE280 electrophoresis chamber. Migration was performed at 120 V under reducing and non-reducing conditions, loading 25 μg of antivenom or 40 μg of venom per lane. After migration, the gels were stained with Coomassie Brilliant Blue G-250, and excess dye was washed with a solution of 10% acetic acid and 10% methanol. Standard molecular weight markers (AccuRuler RGB PLUS Prestained Protein Ladder, MAESTROGEN) were used as reference.

### Affinity chromatography quantification of specific F(ab’)_2_ in antivenoms against venoms

Venoms of *Bothrops asper*, *Crotalus mictlantecuhtli*, and *C*. *basiliscus* were coupled to eight sets of three separate columns with CNBr-Activated Sepharose 4B (CNBrS) from Sigma-Aldrich with modifications to a methodology proposed before [28] to achieve 9 mg in 1.5 mL of the resin. Columns of 1.5 mL were incubated at room temperature for one hour, with up-down mixing, with 20 mg of each antivenom batch. F(ab’)_2_ fragments that did not recognize the resin-coupled venom were washed with PBS (phosphate-buffered saline: 137 mM NaCl, 2.7 mM KCl, 10 mM Na_2_HPO_4_, 1.8 mM KH_2_PO_4_, pH 7.2). Retained F(ab’)_2_ fragments were eluted in two steps, the first one with 5 mL of 0.1 M acetic acid and 10 mL of PBS and the second one using 1 mL of 50 mM NaOH and 7 mL of PBS. The fractions were quantified by measuring absorbance at 280 nm. F(ab’)_2_ fragments detached under basic conditions using 50 mM alkali exhibit very high affinity since they cannot be detached with acid.

### Venom components recognized by antivenoms using affinity chromatography followed by RP-HPLC

Eight mg of the various batches of antivenoms were covalently immobilized to 700 μL of CNBrS and incubated for 1 hour at room temperature with 300 μg of *B*. *asper*, *C*. *atrox*, or *C*. *mictlantecuhtli* venom in 400 μL of PBS with up-down mixing (one column for each venom). Venom molecules that were not retained on the column were recovered using PBS. Subsequently, the immunoretained molecules were eluted using 0.1 M acetic acid in 100 μL of 1 M Tris pH 9. Each fraction was then analyzed by RP-HPLC using an Agilent 1260 Infinity II LC System with a Pursuit xRs-C18 250 x 4.6 mm column. The flow rate was set at 1 mL/min. A linear gradient of 0.1% trifluoroacetic acid (TFA) in water (solution A) and acetonitrile + 0.1% TFA (solution B) was applied, starting with 0% B for the initial 5 minutes, followed by 0–15% B in 10 minutes, 15–45% B in 60 minutes, and 45–70% B in 19 minutes. Protein detection was carried out at 214 nm. The resulting chromatographic profiles of the fractions were compared to those obtained from the analysis of 300 μg of untreated venom.

### Western blotting of antivenom affinity columns

To validate the retention of venom in the columns even after the elution treatment observed, a western blot analysis was conducted. SDS-PAGE was performed under reducing conditions. For samples involving CNBr-Activated Sepharose (CNBrS), 40 μL of the matrix was combined with 10 μL of loading buffer, boiled in a water bath for 5 minutes, and centrifuged at 13,000 rpm for 5 minutes. Fifteen μL of the resulting supernatant was loaded into a lane. For samples composed of soluble proteins, 20 μg were loaded. Following the migration, the proteins in the gel were transferred to a nitrocellulose membrane using a semi-wet chamber and Transfer Buffer (39 mM glycine, 48 mM Tris, 0.037% SDS) for 1 hour at a constant current of 400 mA. The membrane was then incubated with TBST Buffer (10 mM Tris-HCl, 150 mM NaCl, 0.05% Tween-20, pH 7.5) containing 5% skim milk for two hours with constant agitation to block non-ligand-containing sites. Subsequently, the membrane was washed three times with TBST Buffer and incubated for one hour with 10 mL of Antivipmyn batch A1 solution (100 μg/mL) in TBST Buffer as the primary antibody. After the incubation period, the membrane was washed three times with TBST Buffer and incubated for one hour with 10 mL of a 1:7000 dilution of Affinity Purified Antibody Peroxidase Labeled Goat anti-horse IgG (KPL) as the secondary antibody in TBST Buffer. Finally, the membrane was rinsed thrice with TBST Buffer and revealed using 1 mL of Zymed brand TMB Ready-to-Use for Immunoblot. The reaction was stopped with distilled water.

### Assessment of biological and biochemical activities

#### Lethal activity

The median lethal doses (LD_50_) were determined by intravenous injection of varying amounts of venom diluted in 200 μL of PBS into groups of three CD-1 mice weighing 18 to 20 g, following the method described before [29,30]. Mortality was recorded 24 hours after inoculation, and LD_50_ values were estimated using a nonlinear sigmoidal dose-response regression model with GraphPad Prism V8.2.1 software. Surviving mice were euthanized by CO_2_ inhalation.

#### Proteolytic activity on azocasein

The proteolytic activity was assessed by modifying a methodology proposed elsewhere [31]. A 10 mg/mL azocasein (Sigma-Aldrich) solution was prepared in 50 mM Tris-HCl, 0.15 M NaCl, and 5 mM CaCl_2_, pH 8.0. Then, 100 μL of azocasein solution was incubated with 20 μg of each venom in a final volume of 125 μL at 37°C for 30 min, using 100 μL of azocasein with 20 μL of PBS as control. The reaction was stopped by adding 200 μL of 5% trichloroacetic acid and homogenizing the mixture. The tubes were then centrifuged at 13,000 rpm for 10 min, and 150 μL of the supernatant was mixed with 150 μL of 0.5 M NaOH in a 96-well microplate. Subsequently, absorbance was measured at 450 nm with a TECAN model SUNRISE microplate reader, and the data were analyzed with Magellan V4.3 software.

#### Fibrinogenolytic activity

The fibrinogenolytic activity was assessed using human fibrinogen, with modifications to a method described elsewhere [21]. Dilutions containing 50 μg of fibrinogen and 10 μg of each venom were prepared, and each sample was incubated at 37°C for 30 minutes. Additionally, SVMP inhibition assays were conducted using EDTA as a chelating agent. Before fibrinogen digestion, the venom samples were incubated at 37°C for 30 minutes with EDTA. In both cases, the reaction volume was adjusted to 50 μL with PBS at pH 7.2. Finally, a 5 μL aliquot was analyzed using SDS-PAGE under reducing conditions.

### Neutralization

#### Neutralization of lethal activity

The different antivenoms’ ability to neutralize the venoms’ lethal activity included in the project was evaluated by intravenous injection in groups of three CD-1 mice. Different volumes of antivenom were incubated with three LD_50_ as established by the Pharmacopoeia of the United Mexican States (FEUM) [32], of each venom in a final volume of 200 μL of PBS for 30 min at 37°C. The survival percentage of each experimental group was recorded 24 hours after inoculation. The mean Effective Dose (ED_50_), defined as the amount of antivenom required to neutralize the lethal activity 3LD_50_ in half of a given population, was estimated employing a nonlinear sigmoidal-type dose-response regression model using GraphPad Prism V8.2.1 software. Surviving mice were euthanized by CO_2_ inhalation.

#### Neutralization of proteolytic activity

We defined the EC_50_ as the antivenom required to neutralize 50% of the proteolytic activity of 20 μg of venom. Different volumes of antivenom were incubated with 20 μg of each venom in a final volume of 200 μL for 30 min at 37°C. Then, 50 μL of each mixture were added in triplicate to 100 μL of a 15 mg/mL azocasein (Sigma-Aldrich) solution prepared in 50 mM Tris-HCl, 0.15 M NaCl and 5 mM CaCl_2_, pH 8.0. The venom-azocasein-antivenom solution was incubated for 30 min at 37°C, and the reaction was stopped by adding 200 μL of 5% trichloroacetic acid and homogenizing the mixture. The tubes were then centrifuged at 13,000 rpm for 10 min, and 150 μL of the supernatant was mixed with 150 μL of 0.5 M NaOH in a 96-well microplate. Subsequently, absorbance was measured at 450 nm with a TECAN model SUNRISE microplate reader, and the data were analyzed with Magellan V4.3 software.

#### Neutralization of fibrinogenolytic activity

Before fibrinogen digestion, 10 μg of venom were incubated with 400 μg of each antivenom at 37°C for 30 min. Subsequently, 50 μg of human fibrinogen was added to this mixture and incubated at 37°C for 40 min. The reaction volume was brought to 50 μL with PBS pH 7.2. After incubation, a 5 μL aliquot was analyzed by SDS-PAGE under reducing conditions.

## Results and discussion

### Protein quantification of antivenoms

On average, the protein content in each vial of Birmex is approximately 3 to 4.5 times higher than that of Inoserp and Antivipmyn, respectively (Table 2). Our findings regarding the protein quantification of Antivipmyn differ from previous reports, where each vial was reported to have a concentration of 60 mg/mL [33]. This discrepancy may be attributed to the continuous improvement of antivenoms, which generates an increasing proportion of immunoglobulin with higher affinity; over time, this results in a reduced amount of protein to meet established regulations. It is important to note that all eight evaluated batches comply with the protein concentration requirements set by the FEUM, with a maximum limit of 100 mg/mL [32]. For clarity, batch IDs are abbreviated using A for Antivipmyn, B for Birmex, and I for Inoserp.

**Table 2:** Results of protein quantification of antivenoms.

Antivenom	ID	Lot	Protein (mg/mL)
Antivipmyn	A1	B-6J-31	4.6 (4.5–4.8)
	A2	B-8K-31	6.0 (5.3–6.6)
	A3	B-8H-34	6.7 (6.4–6.9)
Birmex	B1	FV045A	26.4 (26.0–26.8)
	B2	FV043A	21.8 (20.5–23.2)
	B3	FV044A	28.6 (27.5–29.7)
Inoserp	I1	8805181002	8.0 (7.6–8.3)
	I2	8805181003	7.4 (7.1–7.8)

Values obtained for protein concentration after reconstituting the vials in 10 mL of distilled water. 95% confidence intervals are indicated in parentheses.

### Electrophoretic profile of antivenoms

**Fig 1:**
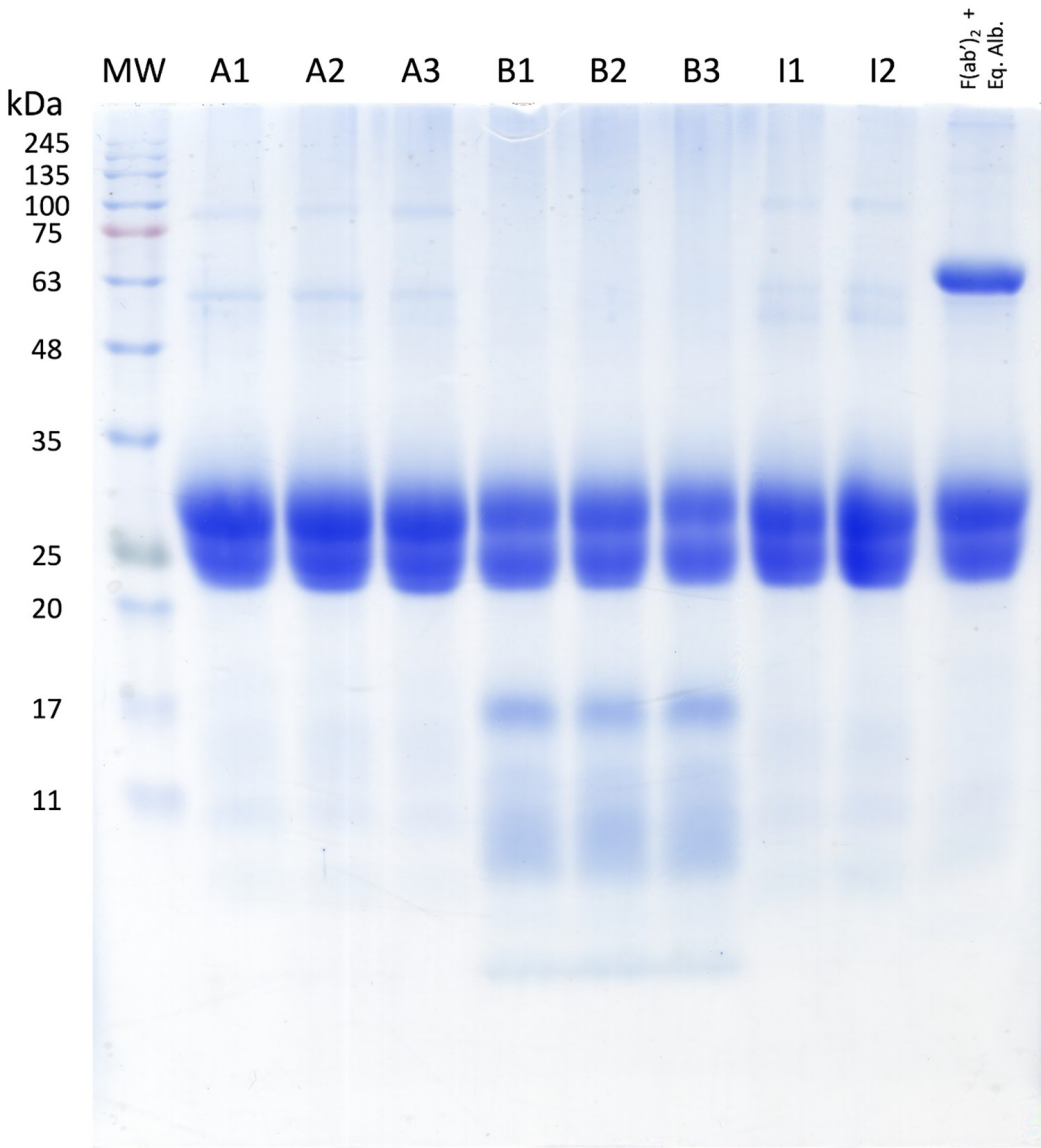
12.5% SDS-PAGE of the antivenoms. Migration was performed under reducing conditions, 25 μg of protein was loaded in each lane, and control with F(ab’)_2_ + equine albumin fragments was included.

Under reducing conditions, two predominant bands of approximately 23 and 27 kDa are observed, corresponding to the expected weight of the light and heavy chains of pepsin-digested IgGs, respectively (Fig 1). The absence of prominent bands at approximately 50 kDa under reducing conditions suggests that all the IgGs have been fully digested or are in meager proportions. However, a western blot would be helpful to confirm this hypothesis, as there have been reports of detecting undigested heavy chains in antivenoms despite their absence in SDS-PAGE assays [34]. Additionally, it is essential to note the presence of bands below 20 kDa in Birmex batches. The identity of these bands remains uncertain in this study, as there are contrasting reports in the literature. In an analysis of different antivenom lots produced by the Butantan and Vital Brazil Institutes, bands of similar weights were identified as protein contaminants unrelated to degradation products of IgGs [34]. However, a different study concluded that bands smaller than 20 kDa in experimental antivenoms represent residues from the digestion of the heavy chain of IgGs and inter-alpha-trypsin inhibitors [35]. These reports emphasize the need for further assays to fully identify the bands migrating below 20 kDa in antivenoms produced by Birmex.

### Quantification of specific F(ab’)_2_

**Fig 2:**
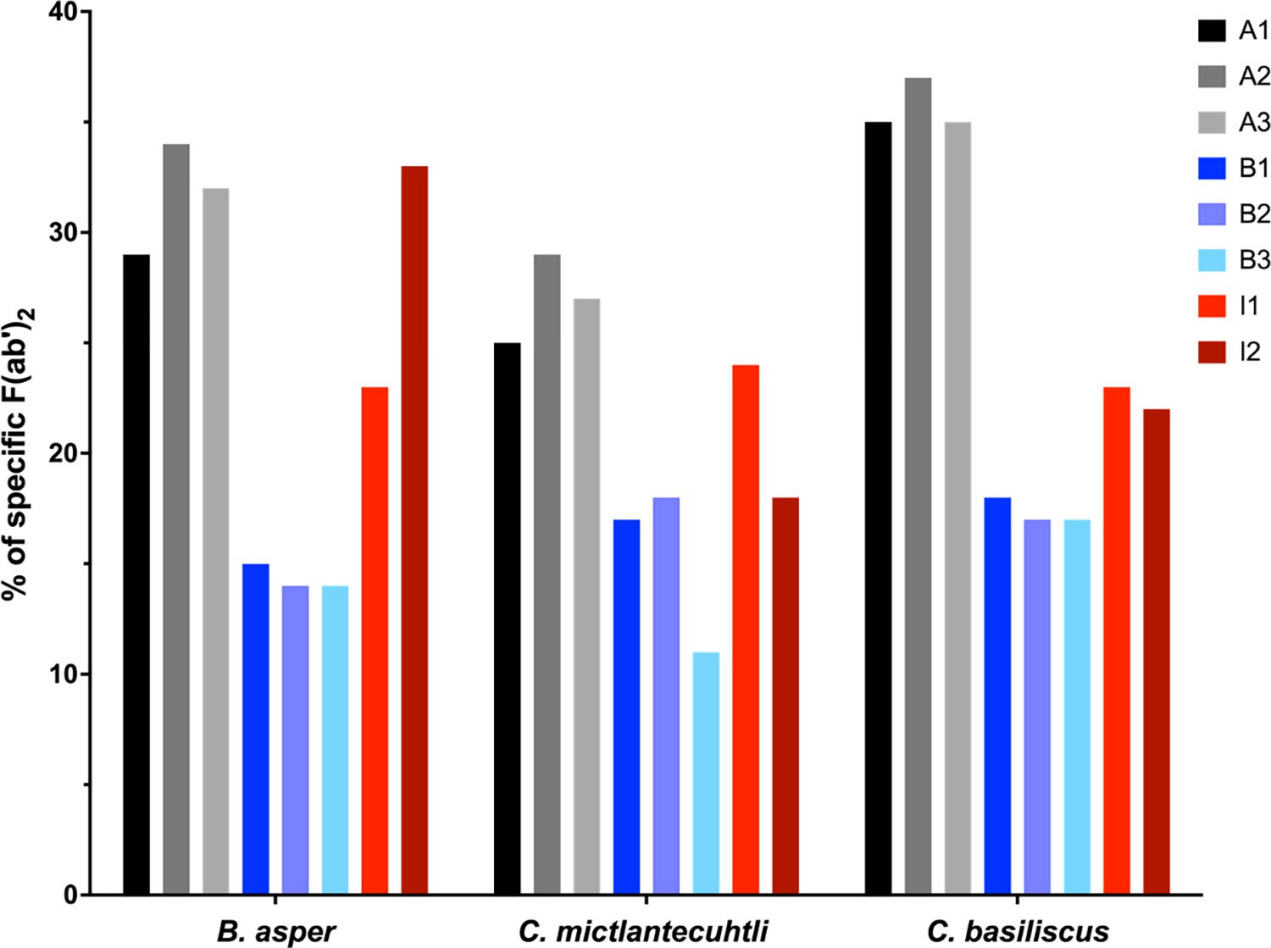
Percentage of specific F(ab’)_2_ fragments contained in the antivenoms.

Quantification of specific F(ab’)_2_ fragments showed that although Antivipmyn has the lowest protein content, it is proportionally the most enriched antivenom in terms of venom-specific antibodies, with on average about twice those contained by Birmex (31% and 16%, respectively) (Fig 2). On the other hand, although it was expected that antivenoms would contain more specific F(ab’)_2_ against the venoms used as immunogens, this was not the case for Antivipmyn, which presented more molecules that recognize the venom of *C*. *basiliscus* (36%), which is not used in its elaboration. This may be explained by the immunological similarity between *C*. *basiliscus* and *C*. *mictlantecuhtli* venom, which produces a strong cross-recognition [36]. Our results agree with what has already been reported for other antivenoms, where only between 5.8 and 36.4% of the total protein content is formed by antibodies (or their fragments) specific against venom [37–39]. Non-specific F(ab’)_2_ fragments could be considered contaminants since they do not participate in the therapeutic effect and may influence the onset of adverse reactions [39].

### Electrophoretic profile of venoms

**Fig 3:**
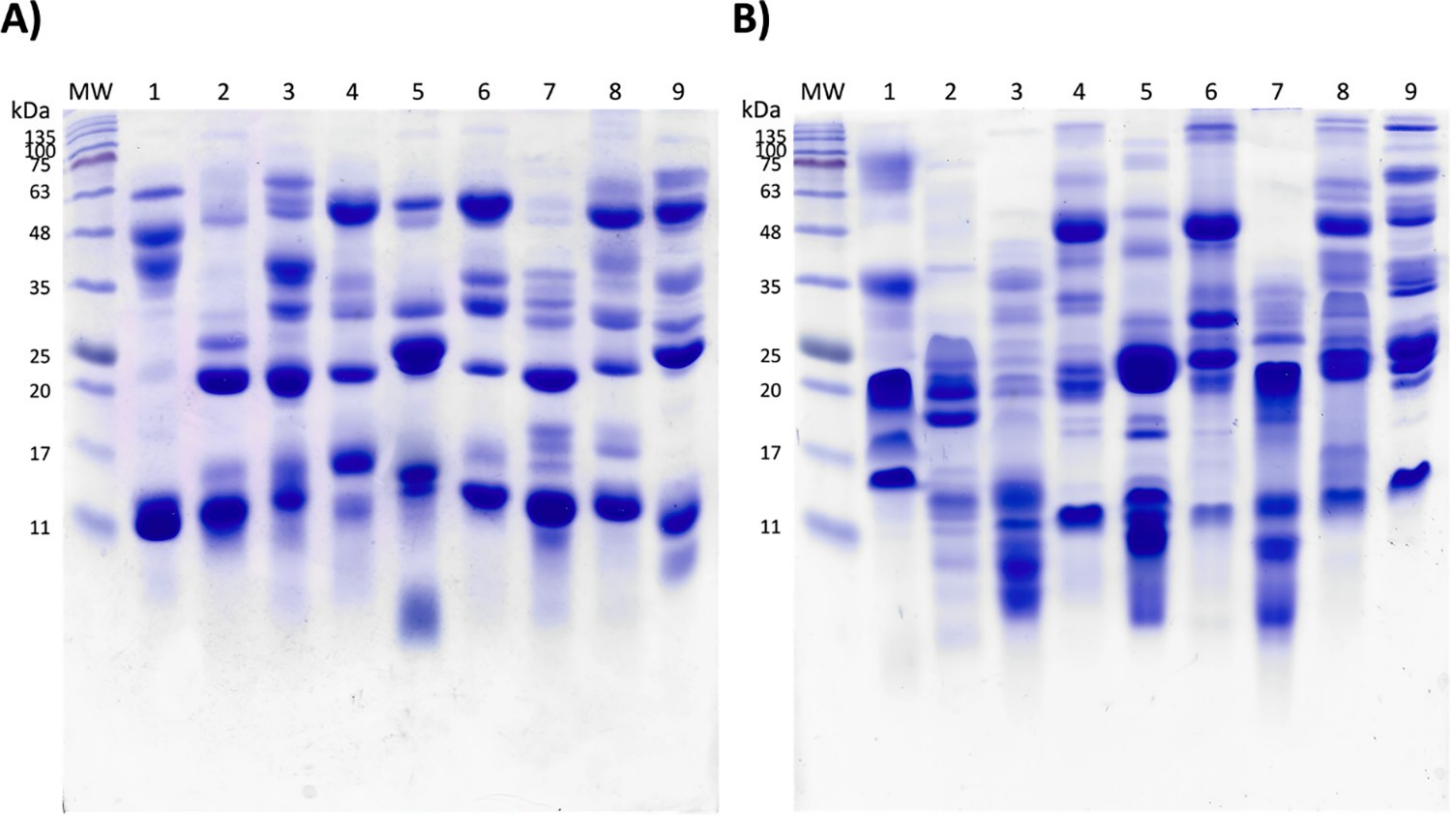
SDS-PAGE of the venoms. A) Reducing conditions; B) Non-reducing conditions. Lane 1, *Agkistrodon bilineatus*; 2, *Bothrops asper*; 3, *Cerrophidion tzotzilorum*; 4, *Crotalus atrox*; 5, *Crotalus basiliscus*; 6, *Crotalus mictlantecuhtli*; 7, *Metlapilcoatlus nummifer*; 8, *Ophryacus sphenophrys*; 9, *Porthidium yucatanicum*.

The electrophoretic profiles of the venoms exhibit significant heterogeneity, displaying a wide range of molecular weight bands with varying intensities (Fig 3). Notably, bands corresponding to 14, 20, and 55 kDa, indicative of PLA_2_, SVSPs, and SVMPs, respectively, are consistently enriched across most venoms. It is important to note that the number of bands observed under SDS-PAGE underestimates the protein diversity within a venom, as many proteins are present in low proportions and may not be visible due to the essay´s sensitivity. Interestingly, while the electrophoretic profiles of venoms belonging to the genus *Crotalus* appear similar under reducing conditions, several differences are observed when analyzing these samples under non-reducing conditions. This highlights the complexity of Mexican viperid venoms, as variations in protein composition exist even within the same genus. Our results prove that Mexican antivenoms face the significant challenge of neutralizing venoms with diverse characteristics.

### Quantification of the recognition of antivenoms to the components of the venoms

**Fig 4:**
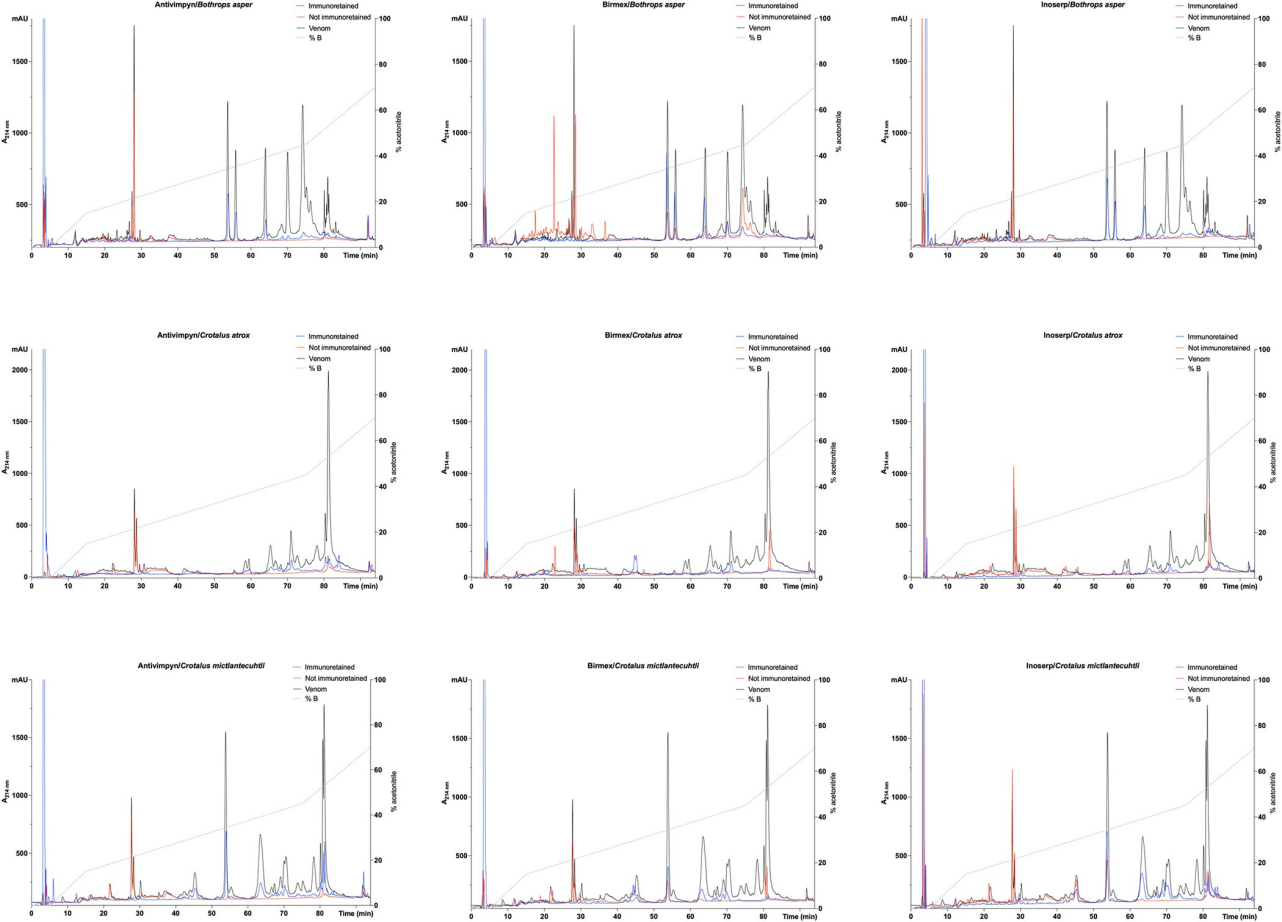
RP-HPLC chromatograms were obtained for the affinity assays. In each graph, 3 chromatograms are shown superimposed: complete venom, recognized fraction, and unrecognized fraction in black, blue, and red, respectively. In addition, the percentage of acetonitrile (B) is shown with a dotted line. The level of recognition toward the venoms of *B*. *asper*, *C*. *atrox*, and *C*. *mictlantecuhtli* was evaluated for Antivipmyn batch A1, Birmex B1, and Inoserp I1.

This test offers a qualitative and quantitative analysis of the venom components recognized by antivenom F(ab’)_2_ fragments. We conducted assays following a logic similar to antivenomics, aiming to identify the venom components recognized to varying degrees. However, it is important to note that we did not perform protein identification through proteomics analysis. It is worth mentioning that proteomic studies have already been conducted for venoms such as *C*. *mictlantecuhtli* [40,41], *O*. *sphenophrys* [42], *M*. *nummifer* [43], and *C*. *basiliscus*, which provides a more precise basis for discussion. One notable observation is that the venoms were not completely recovered, as indicated by comparing the area under the curve of the whole venom with the immunoretained and unrecognized fractions (Figs 4 and S1–S9). If the elution conditions were optimal, the sum of the area under the curve of the immunoretained and unrecognized fractions would be expected to be similar to that of the whole venom. However, in the case of Birmex’s recognition against *B*. *asper* venom, between minutes 15 and 25, two peaks are more abundant in the unrecognized fraction than in the whole venom. We are unaware of these fractions´ identity; however, peptidic and non-protein components, such as disintegrins, tripeptides, and, in many cases, fragments of metalloproteinases, may elute during these times, and these are usually not important for lethality.

Moreover, even with a drastic pH change during elution, the retention of venom molecules on the antivenom column may be attributed to various factors. The pH change induced by the elution buffer may fail to disrupt the antigen-antibody complex interaction entirely. Furthermore, the antibodies used in the study may have been obtained from horses hyperimmunized with the respective venoms over an extended period, leading to a progressive increase in affinity. Additionally, the columns contained many toxins recognized by a diverse range of F(ab’)_2_ fragments with varying specificities, significantly complicating the elution process. The elution conditions for each antigen-antibody interaction are unique and can only be empirically determined [44]. Low recovery percentages have been reported using acidic elution solutions [45]. Conversely, basic eluents were discarded due to evidence of ligand release from CNBrS under slightly alkaline conditions, with the rate increasing as pH rises [46]. This is attributed to the isourea bond between the ligand and CNBrS, which is inherently unstable [47]. It has been suggested that the best approach is to test multiple elution conditions [44]; however, the quantity of antivenom required for such extensive testing poses a technical obstacle. Although there are reports in the literature on dissociation of the antigen-antibody complex using techniques like electrophoresis [48] and increased pressure [49], these techniques were excluded as they may denature proteins and alter the chromatographic profile of the venom. Considering the unrecovered venom as molecules recognized by the antivenom that failed to elute, we found that, on average, SVSPs exhibited the highest recognition rate at 97%, followed by PLA_2_ and SVMPs at 92% and 91%, respectively.

### Western blotting of antivenom affinity columns

**Fig 5:**
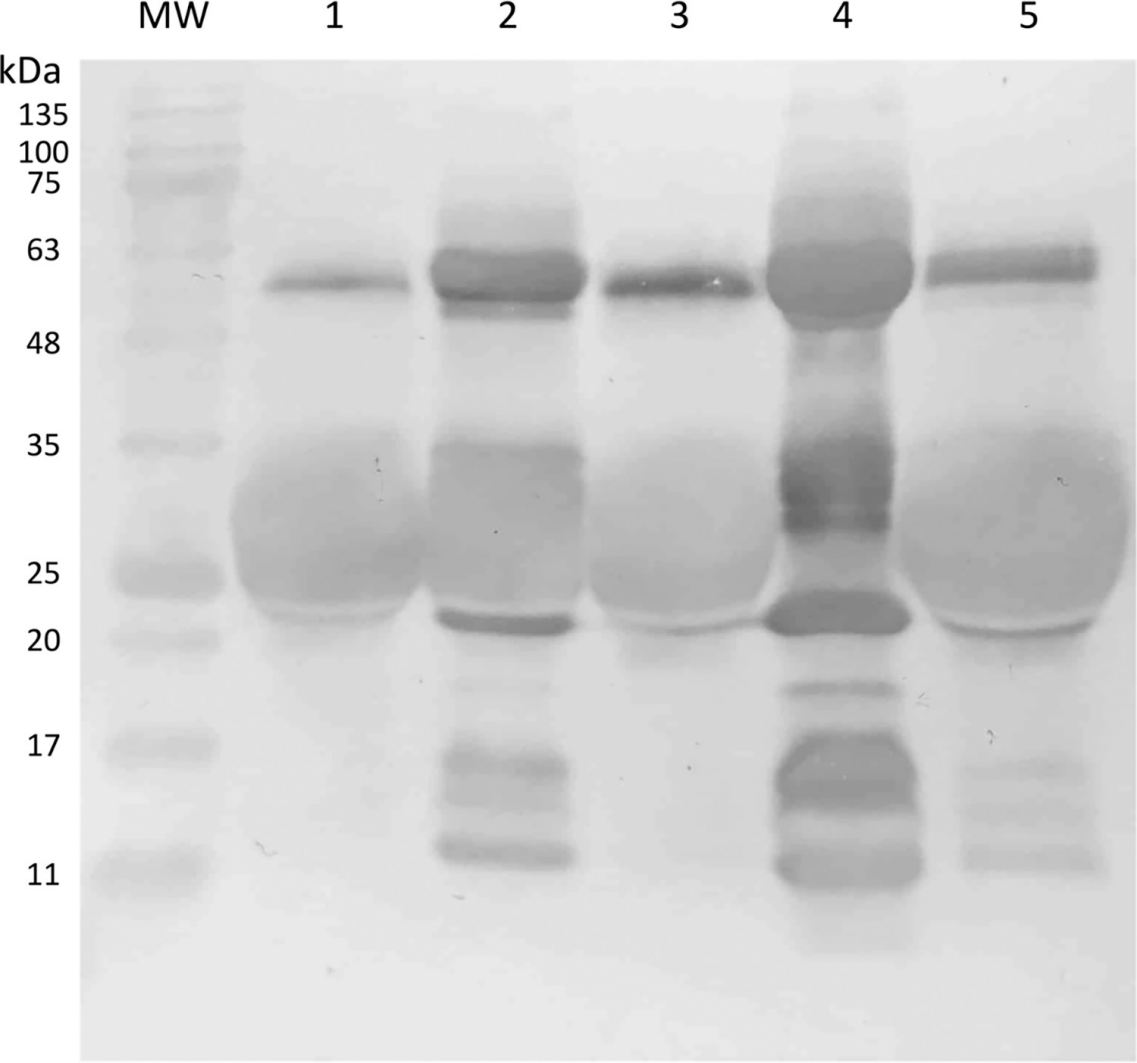
Western blot of the resin where the venom was retained. Lane 1, CNBrS-Antivipmyn batch A1; 2, mixture of *Crotalus atrox* venom and Antivipmyn A1; 3, Antivipmyn A1; 4, *Crotalus atrox* venom; 5, CNBrS- Antivipmyn A1 after incubation with *Crotalus atrox* venom.

The antivenom’s F(ab’)_2_-coupled resin exhibits a clear difference in the banding pattern before and after binding to the venom (Fig 5, lanes 1 and 5). Notably, additional bands in the 10 to 17 kDa range align with bands observed in the venom (Fig 5, lane 4). This observation proves that a portion of the venom remains bound to the CNBrS-Antivipmyn, even after the acid and base elution of the columns.

### Biological and biochemical activities

#### Lethality

The various venoms have a wide range of LD_50_ values, ranging from 0.16 μg/g mouse for *C*. *mictlantecuhtli* to 6.64 μg/g mouse for *M*. *nummifer* venom. As previously reported, the venom of *C*. *mictlantecuhtli*, *C*. *basiliscus*, and *O*. *sphenophrys* induced neurotoxicity [20,41,42,50]. The low LD_50_ further supports this observation compared to other species included in this study (Table 3). We show the raw results in S1 Table.

**Table 3:** Neutralization of lethal activity.

Species	LD_50_	Neutralization (mgAV/mgV)							
	(μg/g)	Antivipmyn			Birmex			Inoserp	
		A1	A2	A3	B1	B2	B3	I1	I2
*A*. *bilineatus*	1.87 (1.84–1.90)	9.89 (9.75–10.04)	8.23 (7.91–8.67)	6.35 (5.94–6.84)	52.94 (52.77–53.13)	47.66 (47.16–48.19)	74.86 (71.32–77.52)	17.04 (16.93–17.14)	19.11 (19.08–19.14)
*B*. *asper*	0.56 (0.54–0.57)	2.41 (2.38–2.43)	3.26 (2.52–3.87)	1.74 (1.64–1.84)	3.10 (2.46–3.88)	5.18 (5.04–5.33)	6.61 (6.35–6.86)	4.60 (4.50–4.72)	3.59 (2.27–5.19)
*C*. *tzotzilorum*	2.53 (2.37–2.72)	7.07 (6.66–7.52)	>18.96*	8.05 (7.07–9.07)	10.79 (9.68–11.91)	13.05 (11.78–14.63)	13.53 (13.03–14.06)	17.67 (17.40–17.95)	>22.57*
*C*. *atrox*	1.99 (1.96–2.02)	11.73 (10.83–12.70)	9.27 (7.53–11.05)	9.92 (8.71–11.33)	19.55 (17.59–21.42)	17.36 (16.52–18.06)	25.94 (25.06–26.51)	15.21 (15.13–15.29)	10.83 (10.82–10.85)
*C*. *basiliscus*	0.75 (0.74–0.75)	4.38 (4.28–4.47)	2.70 (2.54–2.91)	2.84 (2.67–3.06)	6.86 (6.82–6.90)	4.96 (4.31–5.88)	9.09 (8.56–9.60)	4.37 (4.18–4.97)	5.81 (5.49–6.42)
*C*. *mictlantecuhtli*	0.21 (0.18–0.24)	7.53 (6.28–9.31)	13.91 (13.90–13.92)	18.39 (17.14–19.96)	25.85 (25.77–25.92)	20.52 (16.71–26.29)	53.43 (51.84–54.40)	14.54 (14.01–15.06)	29.70 (26.7–30.18)
*M*. *nummifer*	6.90 (6.80–7.10)	>5.42*	>7.32*	>7.70*	>30.89*	>25.79*	>33.87*	>9.41*	>8.71*
*O*. *sphenophrys*	0.88 (0.85–0.90)	15.85 (15.73–15.98)	ND	ND	20.82 (20.78–20.86)	ND	ND	20.97 (20.74–21.20)	ND
*P*. *yucatanicum*	5.46 (5.25–5.67)	>5.63*	ND	ND	>32.04*	ND	ND	>9.76*	ND

Values correspond to the mg of antivenom necessary to neutralize the lethal activity of 1 mg of venom, 95% confidence intervals are indicated in parentheses. *, since the lethal activity was not neutralized even with the highest amount of antivenom administered, the specific neutralization has a higher value than shown; ND, Not determined.

In the case of *C*. *atrox*, our data contrast with the reported LD_50_ from organisms in Veracruz, Mexico [51], which double the value obtained in this study [49]. This discrepancy may be attributed to several factors, such as geographical variation of the venom, the route of inoculation, or the animal’s age. However, the lethality of our pool aligns with the LD_50_ values of *C*. *atrox* venom from the southern USA, which range from 0.94 to 4.3 μg/g, depending on its geographical origin [52]. There are no published data on the venom of Mexican species of the genera *Cerrophidion* and *Porthidium*. The closest taxon for *Cerrophidion* is *C*. *sasai* from Costa Rica [53]. As for *Porthidium*, only the lethal activity of venom from organisms in Colombia, Venezuela, and Costa Rica has been evaluated. An interesting case is the venom of *P*. *lansbergii rozei* from Venezuela, which is not lethal even at a dose of 12.7 μg/g [33,53,54]. It is important to note that in all the studies cited in this paragraph the venom was administered intraperitoneally, which, combined with the species differences, makes direct comparisons challenging.

### Proteolytic activity on azocasein

Our results show differences in the proteolytic activity of the venoms; however, no pattern is observed with respect to the genus to which the species belongs (Table 4). It is worth mentioning that with the exception of *C*. *basiliscus*, the venoms with the highest *in vitro* proteolytic activity correspond to those with higher LD_50_ values, which has been referred to as type 1 or type B venoms [55,56].

**Table 4:** Neutralization of proteolytic activity.

Species	Proteolytic activity	Neutralization ED_50_					
		Antivipmyn		Birmex		Inoserp	
	(U/mg)	A1	A2	B1	B2	I1	I2
*A*. *bilineatus*	1.89 ± 0.11	282.1 (212.4–364.3)	255.1 (213.7–303.8)	*	*	984.9 (855.3–1139.0)	803.4 (752.9–856.4)
*B*. *asper*	1.53 ± 0.03	90.1 (84.5–96.1)	142.5 (126.5–160.3)	105.0 (96.7–114.0)	285.9 (233.8–347.0)	61.8 (50.0–76.3)	190.7 (169.8–214.7)
*C*. *tzotzilorum*	3.02 ± 0.04	ND	ND	ND	ND	ND	ND
*C*. *atrox*	1.83 ± 0.03	283.7 (254.1–316.6)	292.6 (257.8–333.1)	182.0 (156.6–211.6)	845.2 (762.3–948.6)	256.5 (231.6–284.0)	895.6 (807.6–985.6)
*C*. *basiliscus*	3.68 ± 0.09	114.8 (101.3–130.0)	ND	106.3 (94.0–120.3)	ND	230.0 (216.2–244.7)	ND
*C*. *mictlantecuhtli*	1.08 ± 0.20	90.3 (70.0–116.6)	250.2 (214.2–290.8)	362.3 (320.5–409.7)	753.8 (680.4–833.8)	206.0 (184.6–229.9)	513.5 (456.4–573.8)
*M*. *nummifer*	3.02 ± 0.06	599.4 (487.6–736.7)	601.0 (514.4–697.4)	532.0 (487.4–580.7)	775.6 (621.3–979.2)	1068.0 (972.7–1173.0)	1164.0 (999.1–1377.0)
*O*. *sphenophrys*	1.69 ± 0.12	62.47 (52.2–74.8)	ND	279.1 (253.5–307.3)	ND	114.8 (90.1–146.3)	ND
*P*. *yucatanicum*	2.17 ± 0.16	ND	ND	ND	ND	ND	ND

Values correspond to the μg of antivenom required to neutralize 50% of the proteolytic activity produced by 20 μg of venom.

*, the proteolytic activity was not neutralized even with the highest amount of antivenom tested (2,000 μg); ND, Not determined.

#### Fibrinogenolytic activity

All the analyzed venoms have proteolytic activity on the *α*-chain of fibrinogen. In contrast, the venoms of *A. bilineatus* and *C. mictlantecuhtli* partially degrade the β-chain while it is completely degraded by the other venoms. The ɣ chain, on the other hand, is degraded only by the venom of *M*. *nummifer*. There are few records of the degradation of the ɣ-chain of fibrinogen, and all of them were obtained with a higher venom/fibrinogen ratio or with longer reaction times than the conditions used in this work [21,31,57,58]. It should be noted that this is the first report of ɣ-chain degradation by the venom of a snake endemic to Mexico. In the EDTA-treated assays, it is observed that most of the degradation patterns of the fibrinogen chains were modified. In the particular cases of *A*. *bilineatus* and *C*. *basiliscus* where EDTA treatment does not modify the proteolysis of fibrinogen, it can be concluded that SVMPs are not involved in the degradation of this protein, and it is suggested that SVSPs are responsible. A contrasting scenario occurs with the venoms of *O*. *sphenophrys* and *P*. *yucatanicum*, where the digestion of the *α* and β chains is catalyzed mainly by SVMPs, which agrees with what has been reported for the venom of *P*. *lansbergii rozei* [54]. It is also worth mentioning that the degradation of the ɣ chain caused by *M*. *nummifer* venom is caused by SVMPs since the presence of EDTA inhibited it (Table 5).

**Table 5:** *In vitro* fibrinogenolytic activity and its neutralization.

Species	Fibrinogenolytic activity (Chains degraded)										
	Venom	Venom + EDTA	Neutralization								
			Antivipmyn			Birmex		Inoserp			
			A1	A2	A3	B1	B2	B3	I1		I2
*A*. *bilineatus*	*α*	*α*	−	−	−	−	−	−	−		−
*B*. *asper*	*α* & *β*	*α*	−	−	−	−	−	−	−		−
*C*. *tzotzilorum*	*α* & *β*	*α*	*α*	*α*	*α*	*α*	*α*	*α*	*α*		*α*
*C*. *atrox*	*α* & *β*	*α* & *β*	−	−	−	−	−	−	−		−
*C*. *basiliscus*	*α* & *β*	*α* & *β*	−	−	−	−	−	−	−		−
*C*. *mictlantecuhtli*	*α*	−	−	−	−	−	−	−	−		−
*M*. *nummifer*	*α*, *β* & *γ*	*α* & *β*	*α* & *β*	*α* & *β*	*α* & *β*	*α*	*α*	*α* & *β*	*α* & *β*		*α* & *β*
*O*. *sphenophrys*	*α* & *β*	−	*α*	*α*	*α*	*α*	*α*	*α*	*α*		*α*
*P*. *yucatanicum*	*α* & *β*	−	*α*	*α*	*α*	*α*	*α*	*α*	*α*		*α*

The fibrinogen chains degraded under the different treatments are shown. The dash indicates that there is no degradation of any band.

### Neutralization

#### Neutralization of lethality

Sixty-two neutralizations of the lethal activity were performed (Tables 3 and S1), and their specific neutralization values were classified into 5 categories according to the mg of antivenom required to neutralize the lethal activity of 1 mg of venom (mgAV/mgV). The categories were established as follows: well neutralized (ranging from 1.0 to 8.9 mgAV/mgV), medium neutralized (ranging from 9.0 to 16.9 mgAV/mgV), poorly neutralized (ranging from 17.0 to 24.9 mgAV/mgV), very poorly neutralized (above 25 mgAV/mgV), and not neutralized (no neutralization of lethality, even with the highest amount of antivenom tested). Among the venoms tested, the best neutralized were those from *B*. *asper* and *C*. *basiliscus*. All eight lots tested achieved good neutralization of *B*. *asper* venom, while the venom of *C*. *basiliscus* was well neutralized by 7 lots, with B3 showing medium neutralization.

The three lots of Antivipmyn and the two lots of Inoserp exhibited medium neutralization of *C*. *atrox* venom. In comparison, two lots of Birmex poorly neutralized it, and B3 showed very poor neutralization. The venom of *C*. *mictlantecuhtli* was found to be very poorly neutralized by B1, B3, and I2; poorly neutralized by A3 and B2; medium neutralized by A2 and I1, and only A1 showed good neutralization. Regarding *A*. *bilineatus* venom, all three batches of Birmex showed very poor neutralization, the two batches of Inoserp poorly neutralized it, and two batches of Antivipmyn exhibited good neutralization, while A1 showed medium neutralization. On the other hand, no neutralization was observed by A2 or I2 for *C*. *tzotzilorum* venom. However, the remaining two lots of Antivipmyn showed good neutralization, and all three lots of Birmex medium neutralized it, whereas I1 poorly neutralized it. Interestingly, none of the eight tested lots could neutralize *M*. *nummifer* venom. Lastly, *O*. *sphenophrys* and *P*. *yucatanicum* venom were evaluated against one product batch (A1, B1, and I1). The first venom was medium neutralized by A1 but poorly neutralized by B1 and I1. However, none of these three batches could neutralize the lethality of *P*. *yucatanicum* (Table 3).

Regarding the Antivipmyn batches, out of the 23 neutralization tests conducted, 11 (48%) exhibited good neutralization, 6 (26%) showed medium neutralization, one (4%) was classified as bad neutralization, and 5 (22%) did not neutralize even with the highest amount of antivenom tested. For Birmex, which also underwent 23 neutralization tests, 5 (22%) demonstrated good neutralization, 4 (17%) exhibited medium neutralization, another 4 (17%) displayed bad neutralization, 6 (26%) showed very bad neutralization, and in 4 (17%) there was no neutralization observed. In the case of Inoserp, as only 2 lots were tested, a total of 16 trials were conducted, where 4 (25%) showed good neutralization, 3 (19%) displayed medium neutralization, 4 (25%) demonstrated bad neutralization, only one (6%) showed very bad neutralization, and in 4 (25%) there was no neutralization observed.

Theoretically, antivenoms would show better neutralization towards the venoms used as immunogens; however, this was only the case for Birmex. For example, Antivipmyn has a better specific neutralization of *C*. *basiliscus* venom than that of *C*. *mictlantecuhtli*; such cross-reactivity could be explained by the antigenic similarity of these venoms [36]. Inoserp, on the other hand, although it includes *C*. *atrox* venom in its immunization mixture, its specific neutralization is not very different from that of the venoms not used in its preparation. In addition, antivenoms can neutralize (generally with lesser efficacy) some venoms not used as immunogens in their preparation. This cross-neutralization is due to the similarity between the toxins in the venom of phylogenetically related snakes [59]. Previous work has reported the immunogenic deficiency of *C*. *atrox* venom, finding that antibodies produced from this immunogen have lower recognition by the homologous venom compared to those generated from the venom of other species of the genus [60]. Additionally, specific neutralization values of Birmex against *C*. *atrox* venom of 12.8 mgAV/mgV have been obtained [51], which represents double the neutralizing potency for Birmex lot 3 analyzed in the present work, highlighting the variation between lots of the same antivenom.

The venoms not neutralized by the antivenoms were *M*. *nummifer* and *P*. *yucatanicum*. Similarly, two of the eight batches failed to neutralize the lethality of *C*. *tzotzilorum* venom. Although there are no reports of neutralization trials for either *P*. *yucatanicum* or *C*. *tzotzilorum* venoms, the results are contrasting for the case of *M*. *nummifer*. Neutralization of *M*. *nummifer* venom by Antivipmyn injecting five LD_50_ intraperitoneally has been reported [61]; however, in another study, the lethality was not neutralized by Antivipmyn nor Birmex when challenged with two or three LD_50_ injected intravenously [43]. This difference could be explained by the variability in the neutralizing potency of the antivenoms when comparing different batches. In the case of *Porthidium*, the neutralization of *P*. *nasutum* venom from Colombia by Antivipmyn, whose specific neutralization is 8.3 mgAV/mgV, has been reported [33].

We observed a wide variation in the neutralizing potency of the antivenom lots, clear examples being the tests against the venom of *Ophryacus*, *Cerrophidion*, and *Porthidium*. This could be due to various reasons, including the possibility that the plasma mix arises from different horses, changes in both the immunization mix, and in the final protein concentration of each vial may result in a different final product. For this reason, we recommend the use of constant immunogenic mixes to maintain consistent neutralization results.

**Fig 6:**
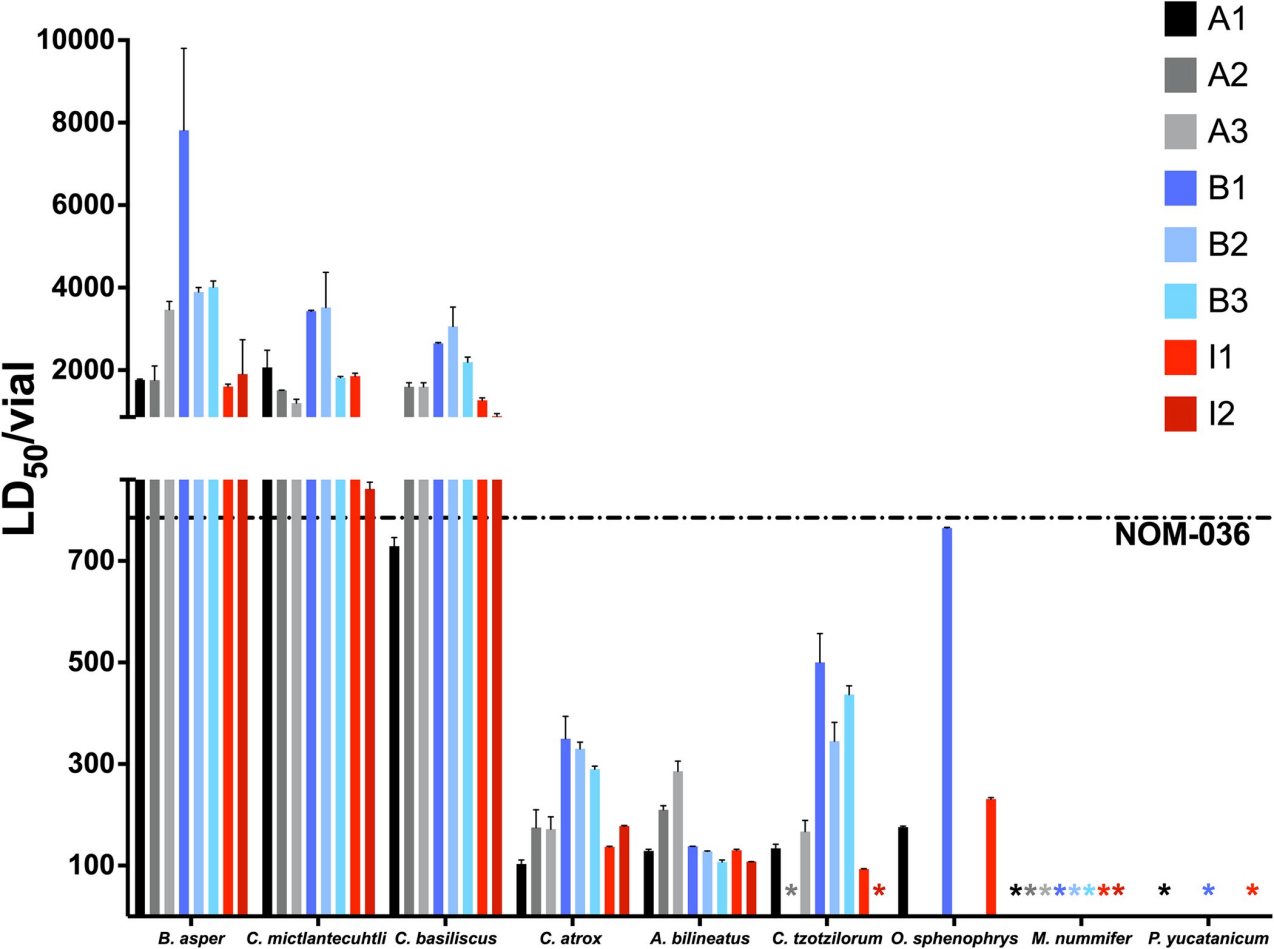
Neutralized LD_50_ values per vial. Error bars correspond to 95% confidence intervals. The dotted line represents the minimum neutralization established by the NOM-036-SSA2-2012. *, the lethal activity was not neutralized even with the highest antivenom administered.

In Mexico, antivenoms are regulated by the Pharmacopoeia of the United Mexican States (FEUM) and the Official Mexican Standard NOM-036-SSA2-2012, which on a per-vial basis establishes "a neutralizing capacity of not less than 790 LD_50_ per mouse of *Crotalus sp*. venom and not less than 780 LD_50_ per mouse of *Bothrops sp*. venom" [62]. To compare our results with the specifications required by NOM-036-SSA2-2012, the neutralizing potencies are also expressed as neutralized LD_50_ per vial (LD_50_/vial) (Fig 6). In this aspect, the analyzed batches of Antivipmyn and Birmex comply with the standard when challenged against the venoms used in their manufacture. The tested batches of Inoserp, which is not currently licensed under NOM-036-SSA2-2012, would not have met the minimum neutralization threshold using *C*. *atrox* venom.

Although species-specific *C*. *atrox* neutralization is not mandated by the FEUM, *C*. *atrox* venom has been broadly studied because of its medical importance in both northern Mexico and the United States [63], and its neutralization is an important basis for comparison across antivenom products. All batches of Inoserp and Antivipmyn showed a medium neutralization of *C*. *atrox* venom on a mgAV/mgV basis, but this is not reflected in the number of neutralized LD_50_, which ranges from 102 to 178 LD_50_/vial. On the other hand, two batches of Birmex neutralized it poorly, and B3 neutralized it very poorly; however, on average, they neutralized more than twice the LD_50_ per vial compared to the other products. In summary, none of the eight batches tested neutralized more than 350 LD_50_ of *C*. *atrox* venom, putting them well below the 790 LD_50_ standard of NOM-036-SSA2-2012. However, this standard does not define the species of the genus *Crotalus* from which venom should be neutralized. The choice of test species used by manufacturers and regulators is essential to releasing commercial lots. However, this leaves the question of whether the standards by genus-level potency support a broad clinical efficacy.

Although preclinical analyses are required to verify the safety and evaluate the effectiveness of an antivenom [64], these studies have physiological limitations because the venom and antivenom injection protocols do not correspond to a real scenario of envenoming. There is even the possibility that the organic response to envenoming/treatment on the part of murine models may differ from that developed by human patients [65]. Although the use of animal models is currently the best tool for analyzing the symptoms associated with poisoning, it is imperative to avoid simplistic extrapolations of these tests to the clinical situation [64].

#### Neutralization of proteolytic activity

The first two batches of each antivenom were evaluated against the venoms of *A*. *bilineatus*, *M*. *nummifer*, *B*. *asper*, *C*. *atrox*, and *C*. *mictlantecuhtli*; for the venoms of *C*. *basiliscus* and *O*. *sphenophrys*, only the first batch of each product was challenged (Table 4). It was impossible to perform these essays with the venoms of *C*. *tzotzilorum* and *P*. *yucatanicum* due to the low availability of venom.

Similar to lethality neutralization, there is variation in the neutralizing capacity of *in vitro* proteolytic activity among different products and between batches of the same antivenom. This is especially evident with the venoms of *B*. *asper*, *C*. *atrox*, and *C*. *mictlantecuhtli*. Furthermore, neither of the two tested batches of Birmex neutralized the proteolytic activity of *A*. *bilineatus* venom, which, along with that of *M*. *nummifer*, was the worst neutralized (Table 4). Although there are no published data on the neutralization of the proteolytic activity on azocasein for the venom of *M*. *nummifer*, the activity of other species of this genus is relatively well neutralized compared to the neutralization of venoms used as immunogens, as *B*. *asper* in the case of the antivenom ICP [66]. This contrasts with our data since there is a large difference in the neutralizing potency of antivenoms towards the different *in vitro* and *in vivo* activities of *B*. *asper* and *M*. *nummifer* venoms.

#### Neutralization of fibrinogenolysis

All batches of antivenoms effectively neutralized the *in vitro* fibrinogenolysis produced by the venom of the genera *Bothrops* and *Crotalus*. However, this did not occur with the venoms of *M*. *nummifer*, *C*. *tzotzilorum*, and *P*. *yucatanicum*, which, even after incubation with the different antivenoms, maintained their proteolytic activity, especially against the α-chain of fibrinogen. The case of *A*. *bilineatus* is interesting because fibrinogenolysis is neutralized by all antivenoms even though the first two batches of Birmex were not able to neutralize their proteolytic activity on azocasein (see above); this suggests that different proteins are involved in the proteolysis of these two substrates, which are differentially neutralized by the antivenoms produced by Birmex (Table 5).

## Conclusions

We found variations in protein content, proportion of specific antibodies, and neutralizing potency against lethal, proteolytic, and fibrinogenolytic activities among different antivenoms and batches of the same product. This suggests the need to homogenize each product’s venom characteristics used as immunogens to avoid batch heterogenicity. In general terms, Birmex can neutralize more LD_50_ per vial due to the amount of protein contained in each vial: 4.5 times more than Antivipmyn and three times more than Inoserp. However, Antivipmyn shows better specific neutralization (mgAV/mgV) against most venoms.

Additionally, our recognition assays show no relationship between recognition and neutralization. The fact that no antivenom neutralized the lethal activity of *M*. *nummifer* and *P*. *yucatanicum* venom, as well as the limited neutralizing capacity of the three antivenoms against the lethal activity of *Agkistrodon bilineatus* and the lack of neutralization of Birmex against the proteolytic activity of *A*. *bilineatus* venom, suggests a significant area for improvement given the medical relevance of these species.

## Supporting information

S1 Fig Recognition level of Antivipmyn A1 toward the venom of *B*. *asper*. Three chromatograms are shown superimposed: complete venom, recognized fraction and unrecognized fraction in black, blue and red, respectively. In addition, the percentage of acetonitrile (B) is shown with a dotted line.
(TIF)

S2 Fig Recognition level of Antivipmyn A1 toward the venom of *C*. *atrox*. Three chromatograms are shown superimposed: complete venom, recognized fraction and unrecognized fraction in black, blue and red, respectively. In addition, the percentage of acetonitrile (B) is shown with a dotted line.
(TIF)

S3 Fig Recognition level of Antivipmyn A1 toward the venom of *C*. *mictlantecuhtli*. Three chromatograms are shown superimposed: complete venom, recognized fraction and unrecognized fraction in black, blue and red, respectively. In addition, the percentage of acetonitrile (B) is shown with a dotted line.
(TIF)

S4 Fig Recognition level of Birmex B1 toward the venom of *B*. *asper*. Three chromatograms are shown superimposed: complete venom, recognized fraction and unrecognized fraction in black, blue and red, respectively. In addition, the percentage of acetonitrile (B) is shown with a dotted line.
(TIF)

S5 Fig Recognition level of Birmex B1 toward the venom of *C*. *atrox*. Three chromatograms are shown superimposed: complete venom, recognized fraction and unrecognized fraction in black, blue and red, respectively. In addition, the percentage of acetonitrile (B) is shown with a dotted line.
(TIF)

S6 Fig Recognition level of Birmex B1 toward the venom of *C*. *mictlantecuhtli*. Three chromatograms are shown superimposed: complete venom, recognized fraction, and unrecognized fraction in black, blue, and red, respectively. In addition, the percentage of acetonitrile (B) is shown with a dotted line.
(TIF)

S7 Fig Recognition level of Inoserp I1 toward the venom of *B*. *asper*. Three chromatograms are shown superimposed: complete venom, recognized fraction, and unrecognized fraction in black, blue, and red, respectively. In addition, the percentage of acetonitrile (B) is shown with a dotted line.
(TIF)

S8 Fig Recognition level of Inoserp I1 toward the venom of *C*. *atrox*. Three chromatograms are shown superimposed: complete venom, recognized fraction, and unrecognized fraction in black, blue, and red, respectively. In addition, the percentage of acetonitrile (B) is shown with a dotted line.
(TIF)

S9 Fig Recognition level of Inoserp I1 toward the venom of *C*. *mictlantecuhtli*. Three chromatograms are shown superimposed: complete venom, recognized fraction, and unrecognized fraction in black, blue, and red, respectively. In addition, the percentage of acetonitrile (B) is shown with a dotted line.
(TIF)

S1 Table Raw data of lethal activity and its neutralization. (XLSX)
