# Supplementary figures and images for: Variability in antivenom neutralization of Mexican viperid snake venoms
Source: PLoS Negl Trop Dis. 2024 May 8;18(5):e0012152. doi: 10.1371/journal.pntd.0012152 (PMC11078402; doi:10.1371/journal.pntd.0012152)

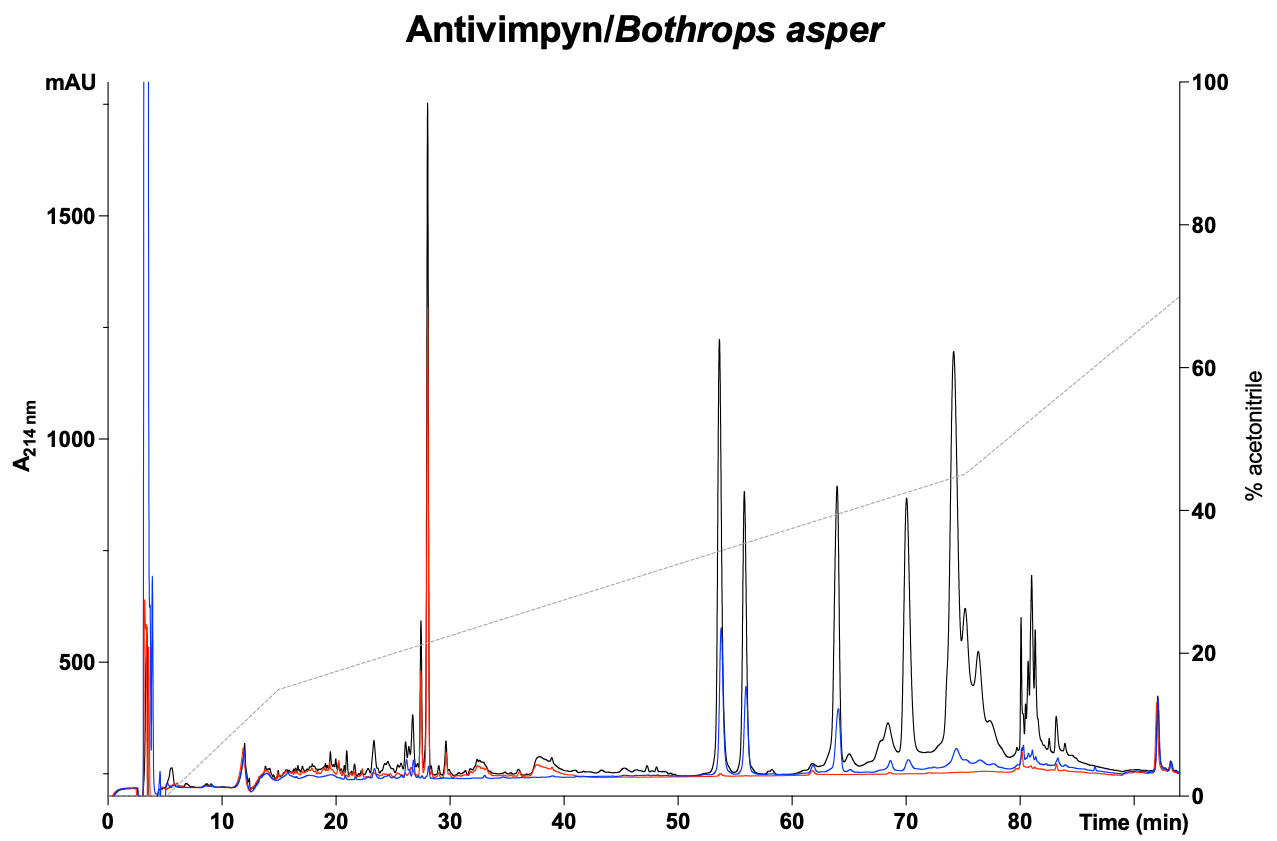

Supplement: S1 Fig — Three chromatograms are shown superimposed: complete venom, recognized fraction and unrecognized fraction in black, blue and red, respectively. In addition, the percentage of acetonitrile (B) is shown with a dotted line. (TIF) [file pntd.0012152.s001.tif]

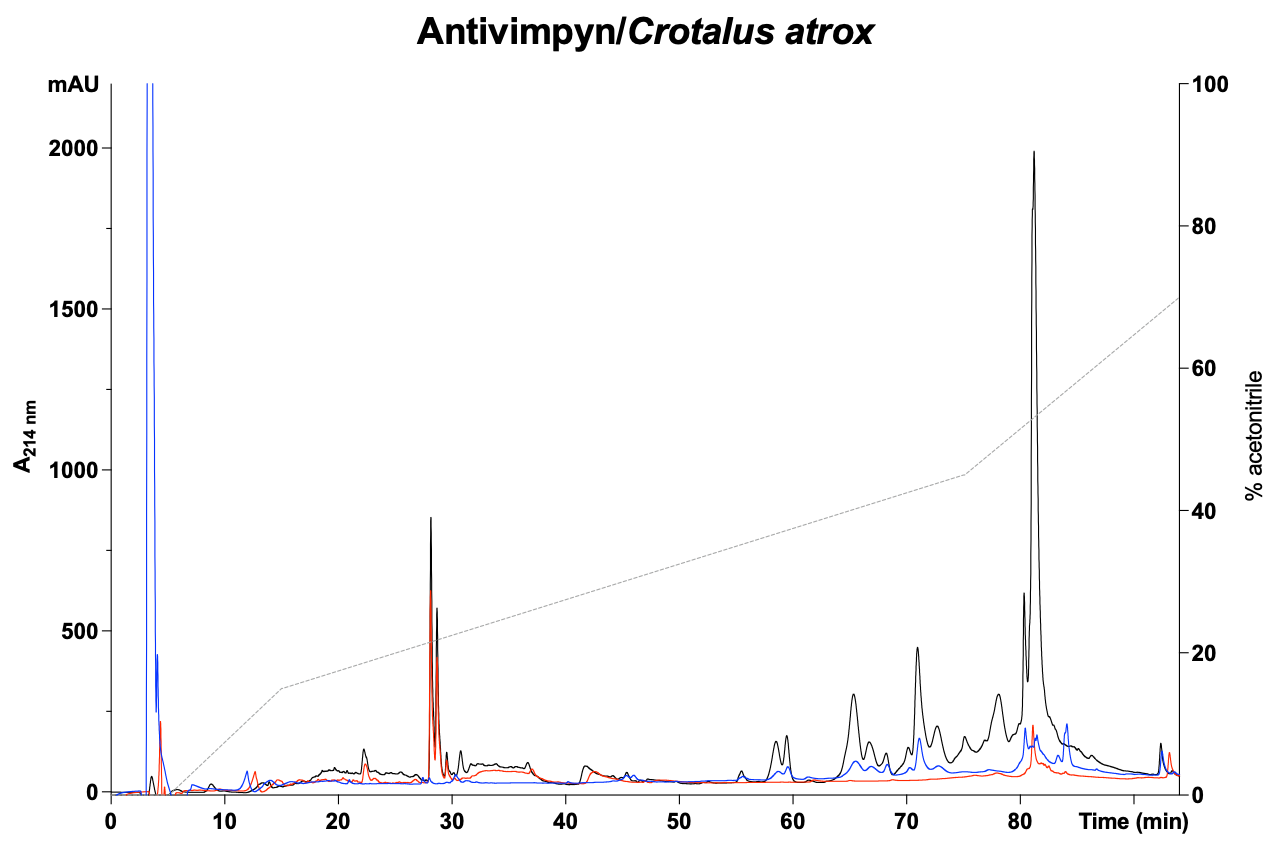

Supplement: S2 Fig — Three chromatograms are shown superimposed: complete venom, recognized fraction and unrecognized fraction in black, blue and red, respectively. In addition, the percentage of acetonitrile (B) is shown with a dotted line. (TIF) [file pntd.0012152.s002.tif]

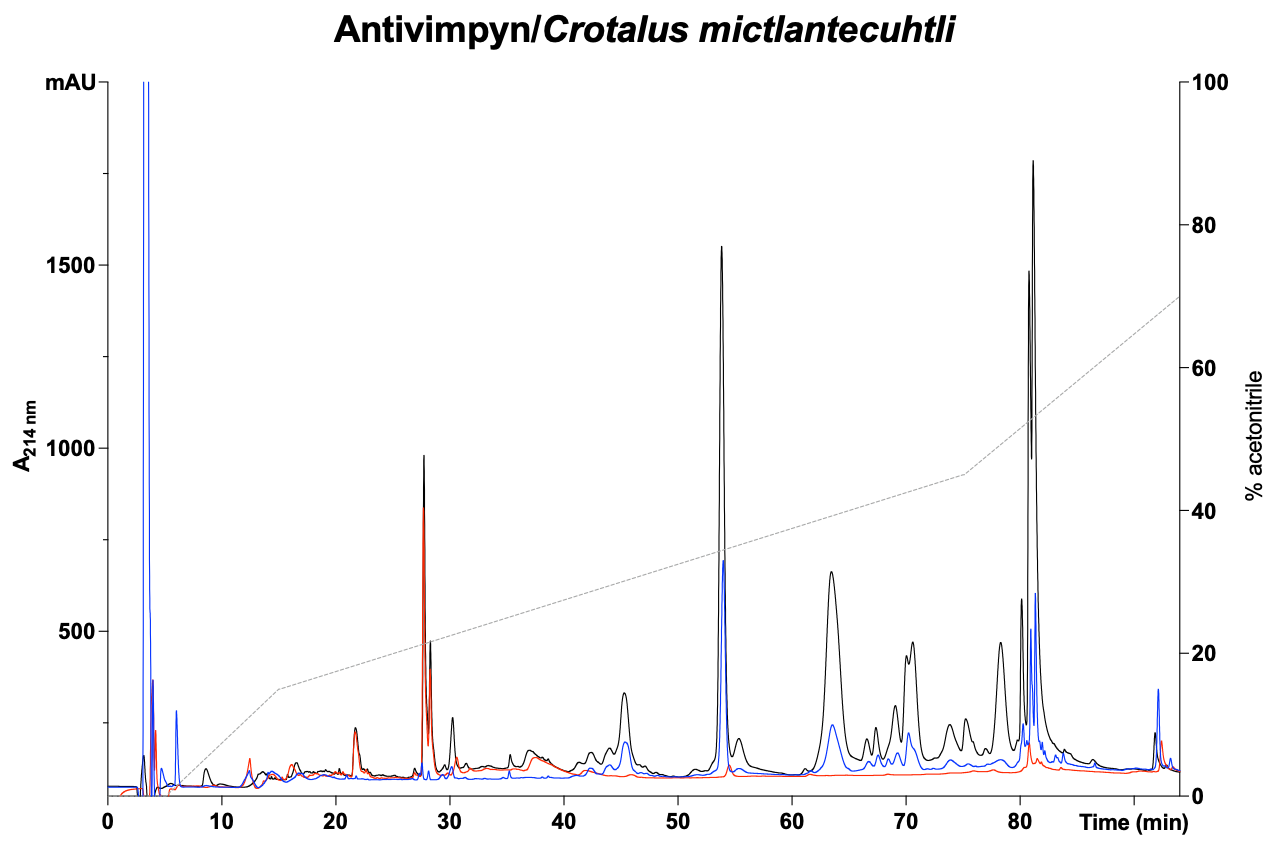

Supplement: S3 Fig — Three chromatograms are shown superimposed: complete venom, recognized fraction and unrecognized fraction in black, blue and red, respectively. In addition, the percentage of acetonitrile (B) is shown with a dotted line. (TIF) [file pntd.0012152.s003.tif]

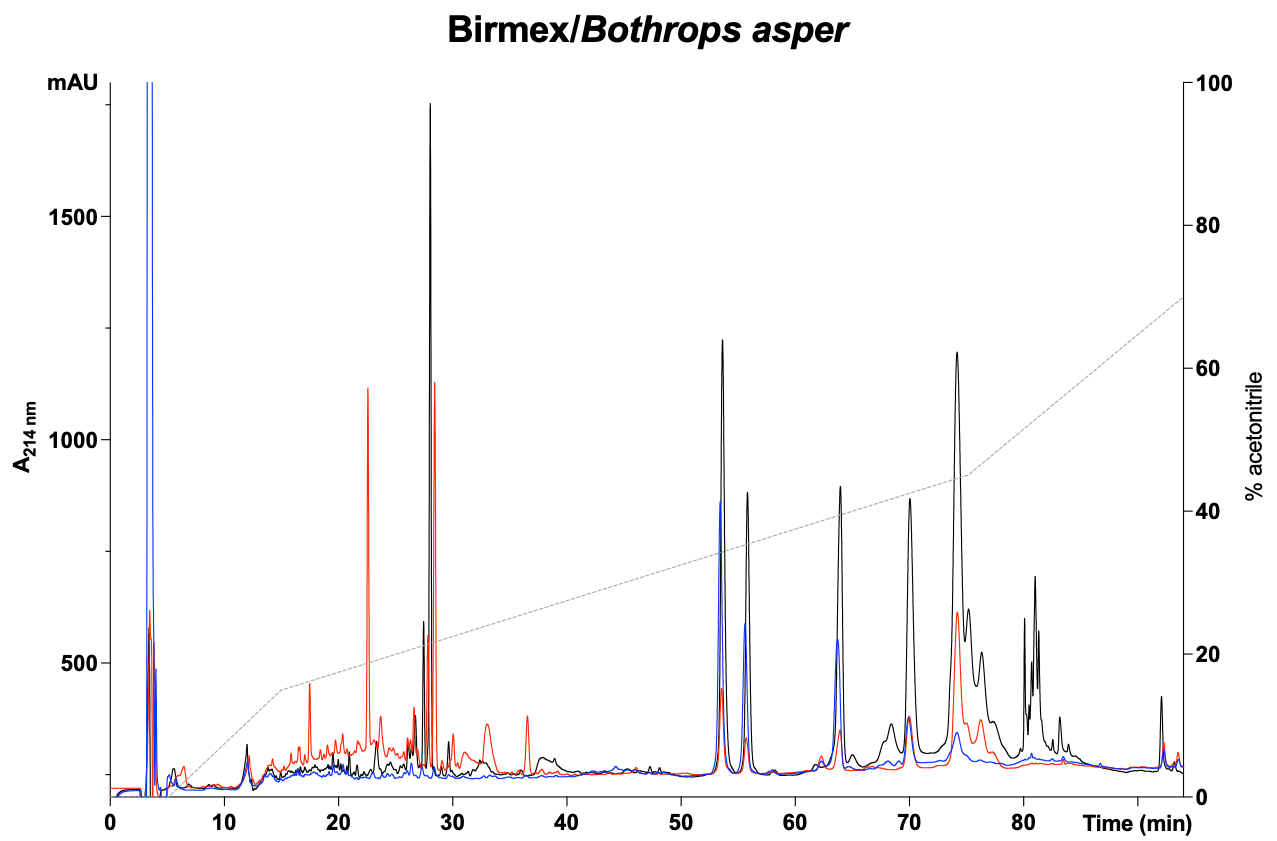

Supplement: S4 Fig — Three chromatograms are shown superimposed: complete venom, recognized fraction and unrecognized fraction in black, blue and red, respectively. In addition, the percentage of acetonitrile (B) is shown with a dotted line. (TIF) [file pntd.0012152.s004.tif]

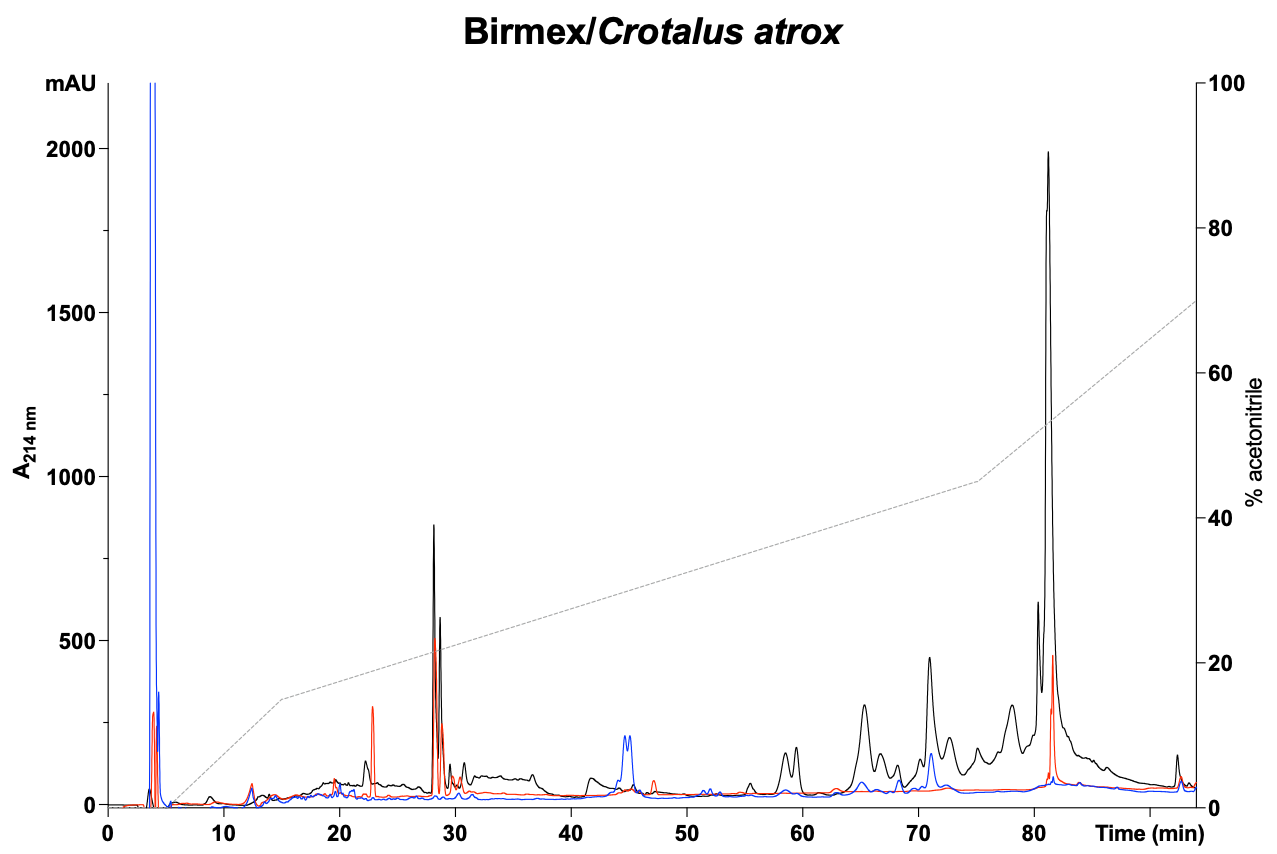

Supplement: S5 Fig — Three chromatograms are shown superimposed: complete venom, recognized fraction and unrecognized fraction in black, blue and red, respectively. In addition, the percentage of acetonitrile (B) is shown with a dotted line. (TIF) [file pntd.0012152.s005.tif]

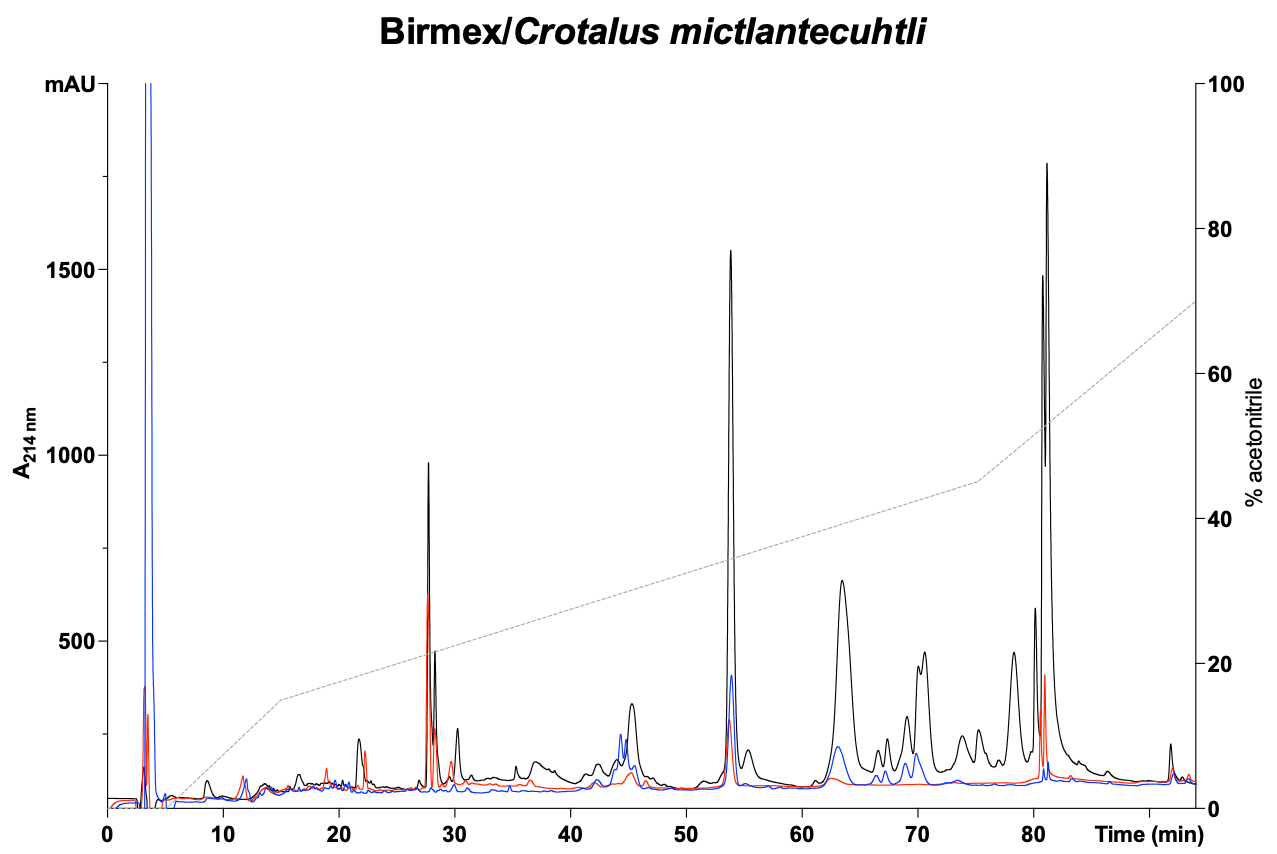

Supplement: S6 Fig — Three chromatograms are shown superimposed: complete venom, recognized fraction, and unrecognized fraction in black, blue, and red, respectively. In addition, the percentage of acetonitrile (B) is shown with a dotted line. (TIF) [file pntd.0012152.s006.tif]

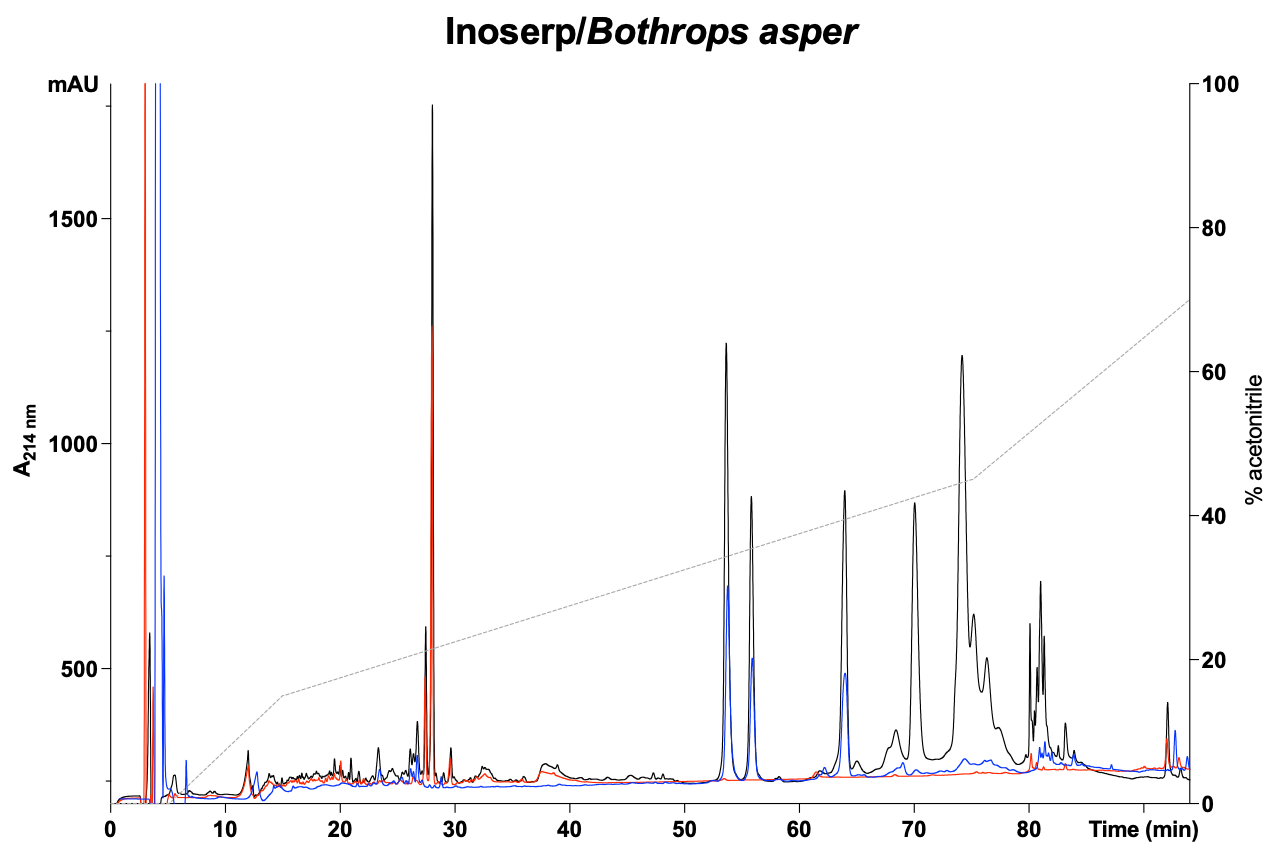

Supplement: S7 Fig — Three chromatograms are shown superimposed: complete venom, recognized fraction, and unrecognized fraction in black, blue, and red, respectively. In addition, the percentage of acetonitrile (B) is shown with a dotted line. (TIF) [file pntd.0012152.s007.tif]

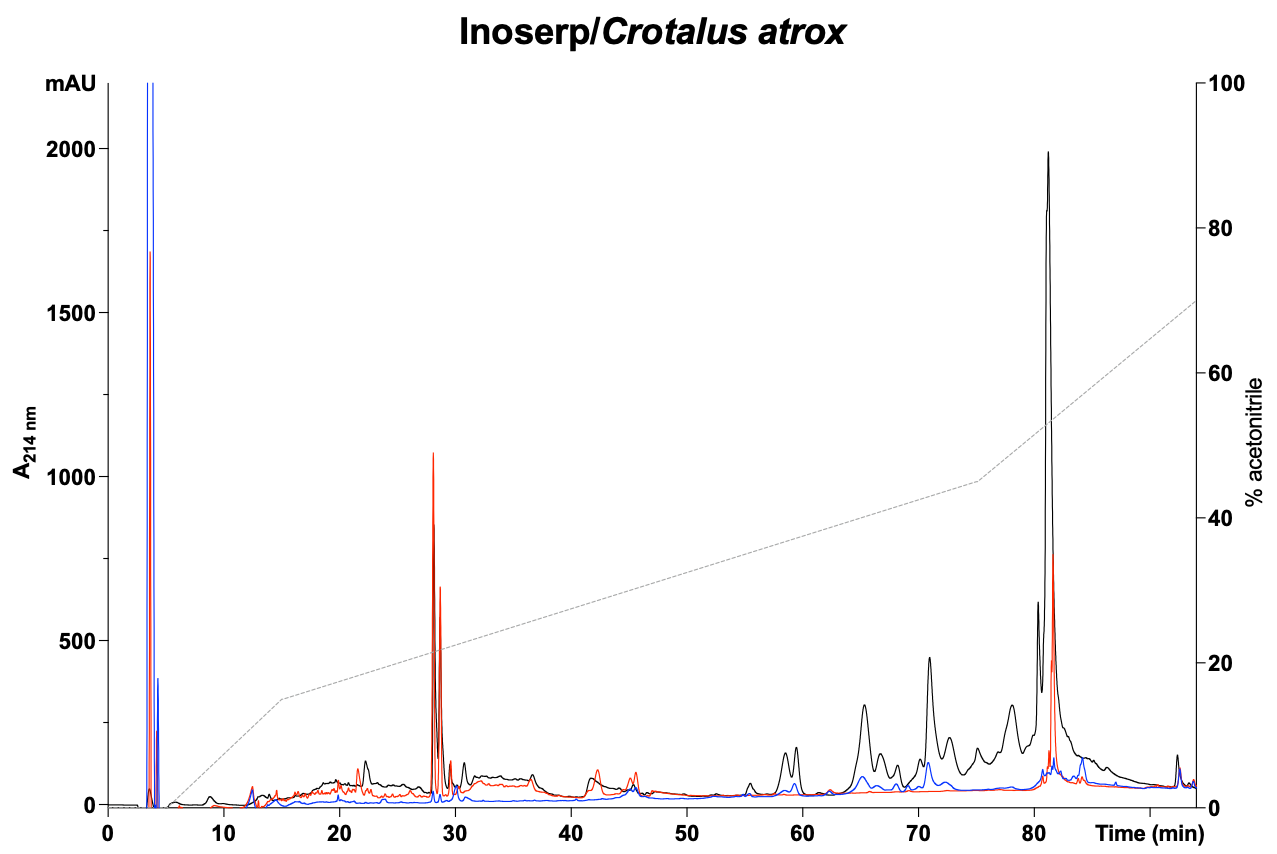

Supplement: S8 Fig — Three chromatograms are shown superimposed: complete venom, recognized fraction, and unrecognized fraction in black, blue, and red, respectively. In addition, the percentage of acetonitrile (B) is shown with a dotted line. (TIF) [file pntd.0012152.s008.tif]

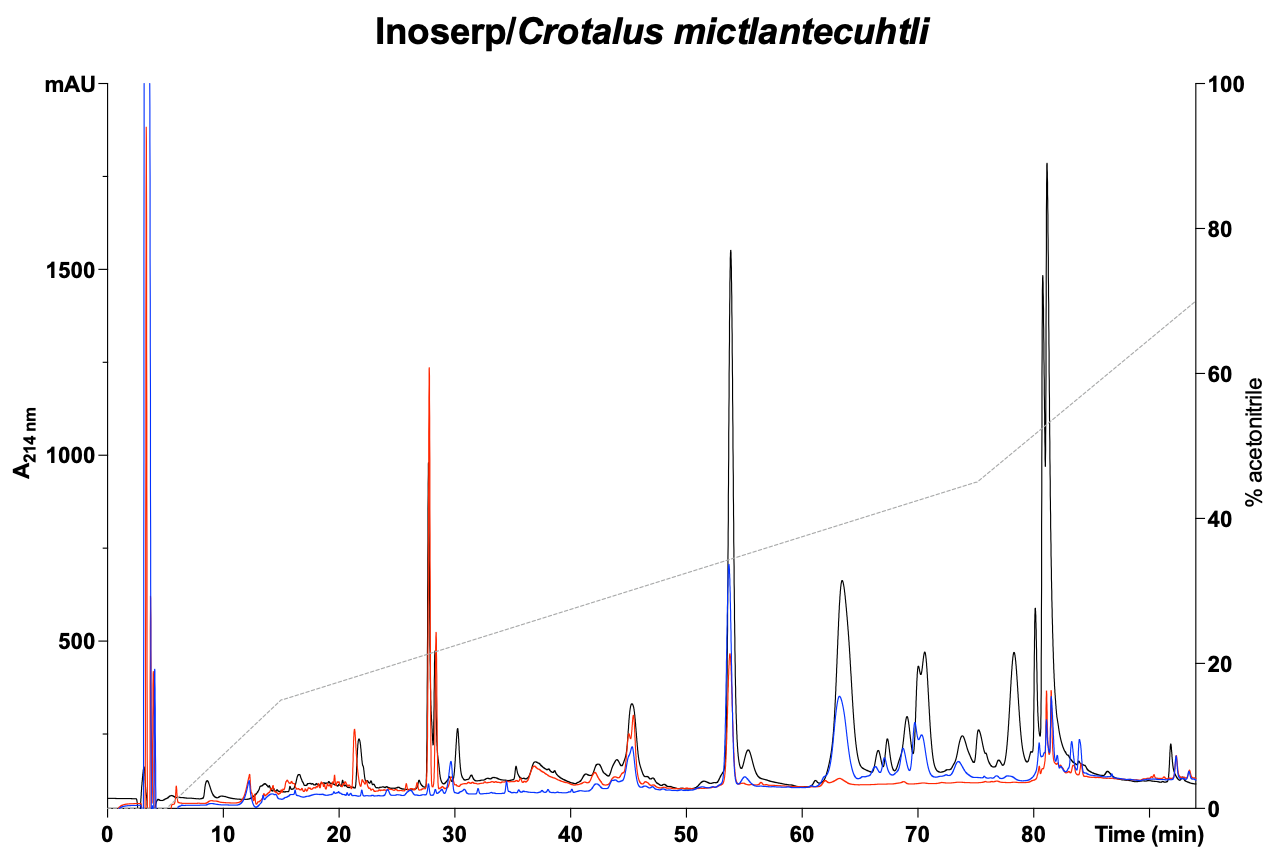

Supplement: S9 Fig — Three chromatograms are shown superimposed: complete venom, recognized fraction, and unrecognized fraction in black, blue, and red, respectively. In addition, the percentage of acetonitrile (B) is shown with a dotted line. (TIF) [file pntd.0012152.s009.tif]
